# Assessment of Evidence for COVID-19 and Rheumatic Diseases: Brief Considerations about Immunity and Pathophysiology

**DOI:** 10.31138/mjr.32.4.287

**Published:** 2021-12-27

**Authors:** Zohreh Jadali

**Affiliations:** Department of Immunology, School of Public Health, Tehran University of Medical Sciences. Tehran, Iran

Severe acute respiratory syndrome coronavirus 2 (SARSCoV-2) is a new strain of coronavirus that has undesirable consequences for people’s livelihoods, national or international economies, and global relations. This novel coronavirus which originated in China, has rapidly spread throughout the world, and was officially considered by the World Health Organization as a global pandemic.

Although SARS-CoV-2 primarily infects the respiratory system, it has a broad potential for extrapulmonary spread and multiorgan involvement. Patients with COVID-19 infection experience a variety of symptoms. The complaints of myalgia or generalised weakness are among the most common problems and could serve as an indicator of disease severity. Moreover, multiple rheumatic manifestations such as arthritis have been reported in COVID-19 patients in various studies. The involvement of the musculoskeletal system including skeletal muscle, bone, and joints have also been reported in infected patients.^1^ Coronavirus can cause complications such as interstitial pneumonia, myocarditis, leukopenia (with lymphopenia), and thrombocytopenia, that may also manifest in rheumatic diseases like systemic lupus erythematosus (SLE) and Sjögren’s syndrome (SS). Neurological symptoms that affect motor control and muscle function have also been reported in up to 36% of patients.^2^

There is also growing evidence for the adverse effect of SARS-CoV-2 infection on endothelial cell function which may contribute to disease pathogenesis by different pathways such as alteration the integrity of vessel barrier, promotion of pro-coagulant activity, induction of inflammation in endothelial cells, and infiltration of leukocytes.^3,4^ Endothelial dysfunction is a very important indicator not only of COVID-19 severity but also of its significance as a common associated problem in a range of immune-mediated inflammatory disorders such as Behçet’s disease.^5^ Therefore, COVID-19 may increase the development and severity of BD or may even trigger BD-like symptoms in susceptible individuals.

In addition to clinical characteristics, the presence of antibodies against nuclear material, anti-SSA (52 or 60 kD) and antiphospholipid antibodies have been reported in patients with COVID-19.^1^ It must be mentioned that these antibodies are associated with some autoimmune rheumatic disorders, such as SLE and SS, and have been used as a valuable diagnostic marker for these disorders. Increased creatine kinase levels (an indirect marker of skeletal-muscle damage) have been also reported in patients with COVID-19.^6^

The abovementioned findings provide preliminary evidence for an association of SARS-CoV-2 infection with rheumatic diseases and are in line with several studies which indicate a causal link between rheumatic disorders and a viral trigger. The benefits of anti-rheumatic drugs in COVID-19 treatment provide another reason for considering the potential link between rheumatology and COVID-19.^7^

Fortunately, rheumatologists quickly understood their responsibility in regards to tackling the COVID-19 pandemic, and their community has created a global alliance with the aim of establishing a registration system for COVID-19 infected patients who suffer from rheumatic diseases.^8^

Patient information can be used for identification of host response mechanisms and development of treatment strategies. However, there are many challenges in understanding factors or relationships that exacerbate or initiate the rheumatic manifestations in patients with COVID-19. The complex nature of rheumatic disorders that can involve different organ system in the body is one of the most important challenges. The exact mechanisms of many rheumatic disorders are still elusive, and multiple risk factors such as gene, environment, and autoimmunity may influence the pathogenesis of these diseases. Variation in virus effects on host and biological complexity of this newly identified virus create another important challenge in understanding the pathogenic mechanisms of COVID-19.

To date, it is difficult to determine whether rheumatic symptoms are reflective of direct or indirect effects of infection. Recent findings indicate that both direct and indirect pathways are important for the onset or aggravation of rheumatic disorders. Direct effects may operate through different mechanisms and depends on dynamic virus–host interactions within a permissive cell. Angiotensin-converting enzyme 2 (ACE2) is an essential host receptor for invasion of SARS-CoV-2 that physically interact with viral spike protein. ACE2 is expressed in various human tissues and its expression has also been documented in several types of musculoskeletal cells, smooth muscles, and synovial tissues. Therefore, the interaction of receptor-ligand may be the basis for the cytopathic effects of virus. However, it is not clear whether the virus can directly infect musculoskeletal tissues.^6^

In addition to direct mechanisms, adverse rheumatologic consequences may occur as a result of indirect mechanisms. For instance, some evidence indicates that the immune-mediated response against the virus can indirectly affect different organs.

Cytokine storm and excessive production of inflammatory mediators is one of the major causes of multiple-organ failure in COVID*-*19 infections. Cytokines are important for host defence, but their uncontrolled production may lead to pathological changes in different organs such as skeletal muscle tissue. It has been shown that IFN-y, IL-1β, IL-6, IL-17, and TNF-α can directly promote proteolysis of muscle fibre and downregulate protein synthesis. Moreover, IL-1β and TNF-α can block the proliferation and differentiation of Satellite cells that are responsible for muscle growth. Additionally, the potential role of IL-1β and IL-6 in the fibrosis process could impair the production of muscular force and can increase the vulnerability of patients to injury.

In COVID-19 patients, the effect of immune cytokines on bone is another important aspect for consideration. CXCL10, IL-17, and TNF-α are three important cytokines that are produced in response to SARS-CoV-2 infection and play a key role in bone metabolism. These three cytokines act on the osteoclasts and can result in inadequate bone mineralization by inducing osteoclastogenesis or by decreasing osteoblast proliferation and differentiation. The role of cytokines in joint pathology and tendinopathy also should not be disregarded.^1^

Counteraction of protective effects of ACE2 in musculo-skeletal system by SARS-CoV-2 can also be considered as an alternate mechanism of disease pathogenesis.^9^

In summary, the SARS-CoV-2 can invade the musculoskeletal system and can initiate different rheumato-logic symptoms by a variety of direct or indirect mechanisms. Therefore, close monitoring of patients with rheumatic disorders and their response to treatment play significant role in patient care.

## Abbreviations

Angiotensin-converting enzyme 2: (ACE2)
Severe acute respiratory syndrome coronavirus 2: (SARS-CoV-2)
Sjögren’s syndrome: (SS)
Systemic lupus erythematosus: (SLE)
